# Retrospective Frailty Assessment in Older Adults Using Inertial Measurement Unit-Based Deep Learning on Gait Spectrograms

**DOI:** 10.3390/s25113351

**Published:** 2025-05-26

**Authors:** Julius Griškevičius, Kristina Daunoravičienė, Liudvikas Petrauskas, Andrius Apšega, Vidmantas Alekna

**Affiliations:** 1 Department of Biomechanical Engineering, Vilnius Gediminas Technical University, LT-10105 Vilnius, Lithuania; julius.griskevicius@vilniustech.lt (J.G.);; 2 Faculty of Medicine, Vilnius University, LT-03101 Vilnius, Lithuania; andrius.apsega@mf.vu.lt (A.A.);

**Keywords:** frailty, IMU, spectrogram, convolutional neural networks, classification, gait

## Abstract

Frailty is a common syndrome in the elderly, marked by an increased risk of negative health outcomes such as falls, disability and death. It is important to detect frailty early and accurately to apply timely interventions that can improve health results in older adults. Traditional evaluation methods often depend on subjective evaluations and clinical opinions, which might lack consistency. This research uses deep learning to classify frailty from spectrograms based on IMU data collected during gait analysis. The study retrospectively analyzed an existing IMU dataset. Gait data were categorized into Frail, PreFrail, and NoFrail groups based on clinical criteria. Six IMUs were placed on lower extremity segments to collect motion data during walking activities. The raw signals from accelerometers and gyroscopes were converted into time–frequency spectrograms. A convolutional neural network (CNN) trained solely on raw IMU-derived spectrograms achieved 71.4 % subject-wise accuracy in distinguishing frailty levels. Minimal preprocessing did not improve subject-wise performance, suggesting that the raw time–frequency representation retains the most salient gait cues. These findings suggest that wearable sensor technology combined with deep learning provides a robust, objective tool for frailty assessment, offering potential for clinical and remote health monitoring applications.

## 1. Introduction

Frailty refers to a clinical syndrome characterized by diminished physiological reserves that increase the risk of adverse health outcomes such as deteriorating mobility, disability, falls, hospital admissions, and death [1]. Although frailty can be reversed, especially in its initial phases [2,3,4], its detection often relies on subjective evaluations, such as Fried’s five criteria (weight loss, exhaustion, inactivity, slowness and weakness) [1]. These criteria may be prone to bias and lack feasibility for routine care, as self-reported measures can be inconsistent [5,6,7,8]. Objective methods for frailty early detection are therefore highly sought after [9,10].

A growing body of research suggests that gait parameters, such as stride time variability, step length and double support time, may offer early markers for frailty [11,12]. However, such studies typically require laboratory-based systems, such as camera systems, force platforms, or computerized walkways [13,14,15], which are often costly and impractical in daily clinical settings. Wearable devices like inertial measurement units (IMUs) provide a promising alternative for continuous gait monitoring in real-world environments, especially combined with recent technological advancements in deep learning algorithms for signal classification [16,17,18]. IMUs can capture acceleration and angular velocity at multiple body segments, potentially revealing subtle mobility alterations prior to clinical manifestations of frailty [19,20,21,22]. Unlike traditional optical motion capture systems that require multiple cameras, studies use one or more IMUs on different body parts to capture gait dynamics. Data from a single IMU are effective for activity recognition via mobile phones or smartwatches [23,24,25,26]. In gait analysis, even a single IMU on each foot can monitor gait asymmetry and other parameters effectively [27,28,29,30]. Using multiple sensors improves frailty evaluation by providing quantitative data to differentiate levels of frailty [31,32].

Although IMUs offer numerous advantages, the analyzing IMU data can be complex, involving robust algorithms for gait events detection and intricate signal processing methods like filtering and noise reduction, which might produce varying outcomes regarding the accuracy of feature extraction [32,33,34,35]. IMU-based human movement analysis can be performed in time and frequency domains. Combining both analyses can enhance understanding of gait characteristics, particularly in pathological conditions like Parkinson’s disease, stroke, or frailty [35,36,37,38].

The integration of machine learning (ML) algorithms and convolution neural network (CNN) techniques by converting IMU time-domain data into time–frequency representations (e.g., spectrograms, Gramian Angular Field GAF, Recurrence Plot and Markov Transition Field images) has further enhanced the predictive accuracy for frailty identification, classifying Parkinson’s disease patients, healthy controls and gymnast actions [36,39,40,41,42,43]. Arshad et al. successfully combined short time Fourier transform (STFT) and GAF representations of IMU gait signals for frailty assessment [44]. These image-based approaches leverage machine vision techniques as an alternative to optical motion capture systems, enabling markerless gait classification through CNNs [36]. Beyond CNNs, other time series classification algorithms (e.g., InceptionTime) have also proven effective for frailty classification, achieving an 81% accuracy on test data [45,46]. Given that frailty datasets are frequently small and heterogenous, there remains a need for optimized strategies to harness IMU data—particularly minimally processed signals—without extensive feature engineering [47,48,49,50,51,52].

This study aims to evaluate the feasibility and effectiveness of using raw or minimally processed IMU-derived spectrograms for frailty classification via deep learning. Unlike prior investigations that rely heavily on custom-engineered features or highly processed signals, we focus on determining whether raw IMU data retains sufficient discriminatory power to differentiate among Frail, PreFrail and NoFrail older adults. Additionally, we compare multiple signal preprocessing approaches (e.g., baseline gravitational offset removal, low-pass filtering) to assess their impact on classification performance. By reducing the need for complex signal processing and leveraging spectrogram-based representations, we seek to streamline frailty assessment for potential clinical and real-world applications, thereby filling the gap in the existing literature where many proposed methods remain resource-intensive or inconsistent across different populations.

## 2. Materials and Methods

This study was conducted retrospectively using an existing IMU dataset originally collected from the study by Apšega et al., which investigated the association between frailty levels and gait parameters derived from wearable sensors [32]. Although the original publication focused on correlational analysis and descriptive statistics, the present work extends those findings by performing a deep learning–based classification of frailty status.

Frailty Gait Database (FRGaitDB) comprises clinical screening data and IMU-based recordings from 133 participants with an average age ± SD of 75.1 ± 8 and a body mass index of 27.6 ± 5.8. The frailty level was categorized following Fried et al. [1]: NoFrail = no criteria; PreFrail = one or two criteria; and Frail = three or more criteria out of the five components, consisting of weight loss, weakness, exhaustion, slowness and low physical activity. Based on this evaluation, participants were assigned to Frailty (*n* = 35), PreFrail (*n* = 67) and NoFrail (*n* = 31) groups. Six wireless inertial measurement units (IMUs, Shimmer Research, Dublin, Ireland) were secured to the thighs, shins and feet using straps. Subjects were instructed to walk 4 m at their normal speed, and data from three separate trials were used for further processing and analysis. The sensors’ data were collected through a Bluetooth wireless connection at a sampling rate of 256 Hz.

**Figure 1:**
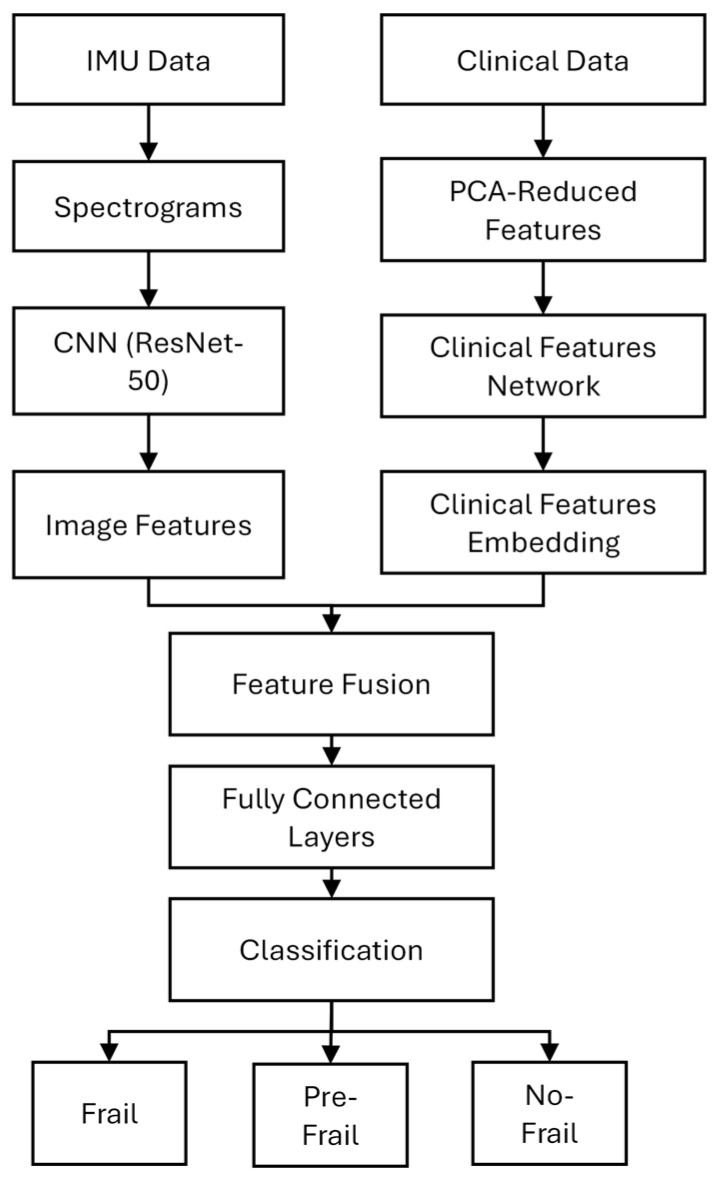
Multimodal deep learning model architecture.

A multimodal deep learning model was developed to classify participants into three frailty categories: Frail, PreFrail, and NoFrail. The data processing and classification model architecture consisted of two parallel branches—image-based and clinical-based— and it is shown in Figure 1.

Data processing steps are described in subsequent sections.

### 2.1. IMU Data Acquisition and Preprocessing

The dataset comprised IMU sensor recordings from participants during gait trials. Each participant had inertial measurement units attached to both lower limbs (thigh, shank and foot). The IMU sensors recorded tri-axial accelerometer and gyroscope data across three walking trials per participant.

#### 2.1.1. IMU Data Preprocessing and Spectrogram Generation

**Figure 2:**
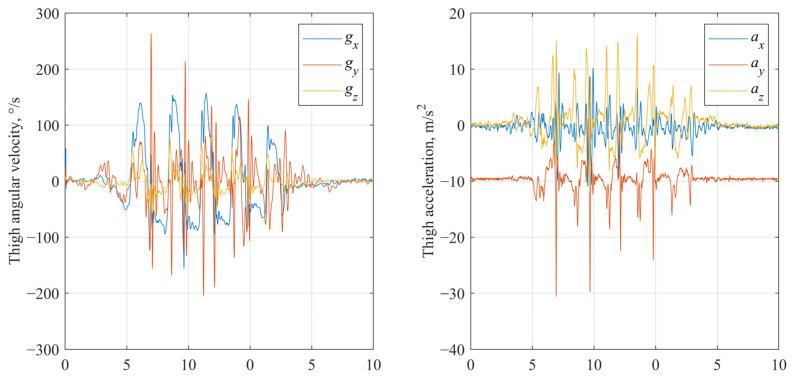
Raw gyroscope and accelerometer signals of IMU fixed on thigh segment.

**Figure 3:**
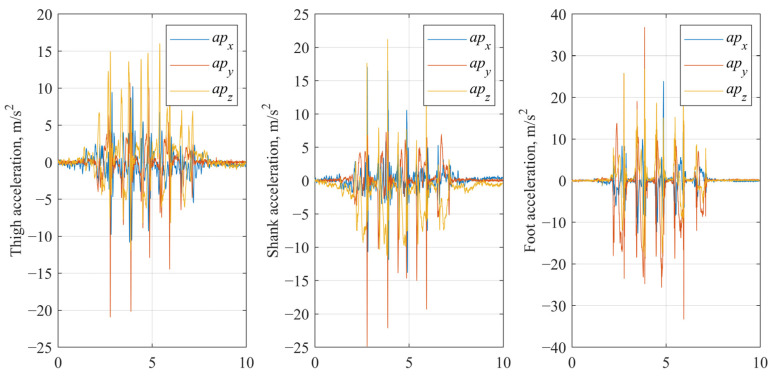
Baseline processed accelerometer signals of IMU fixed on thigh, shank and foot segments.

**Figure 4:**
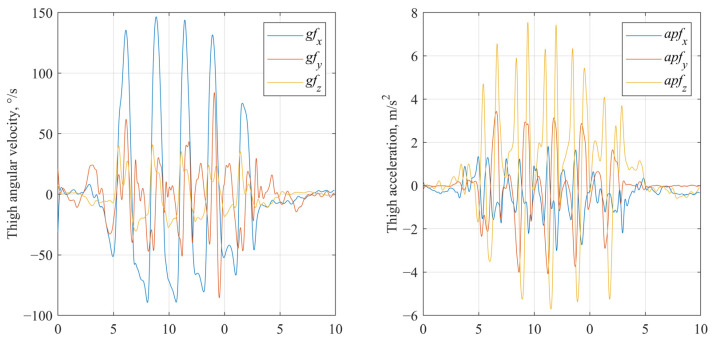
Low pass filtered gyroscope and accelerometer signals of IMU fixed on thigh segment.

The data were processed into three distinct datasets to evaluate the impact of preprocessing on classification performance:

- Raw signals (no processing). Unaltered tri-axial accelerometer and gyroscope signals were used to evaluate the baseline capability of the model to learn directly from unfiltered sensor data. Examples of raw accelerometer and gyroscope signal are shown in Figure 2.

- Preprocessed signals (baseline processing). In the baseline processing, accelerometer data were detrended to remove gravitational offset [50]. For each gait trial, a baseline gravitational component was estimated as the mean acceleration over samples 5 to 30, corresponding to an initial standing phase, which was then subtracted from the entire accelerometer signal along each axis, effectively isolating dynamic acceleration related to movement (Figure 3). Gyroscope data were retained in their original form.

- Filtered signals (low pass filtering). The gravity-corrected accelerometer signals (from the baseline processed dataset) and raw gyroscope signals were further processed using a 2nd-order low-pass Butterworth filter with a cut-off frequency of 8 Hz, applied separately to each axis (Figure 4). The selection of the 8 Hz cutoff frequency was informed by the existing literature and gait frequency analysis. Typical human walking involves frequency components predominantly below 10 Hz, with higher frequencies generally attributed to noise or extraneous motion artifacts [52,53].

**Figure 5:**
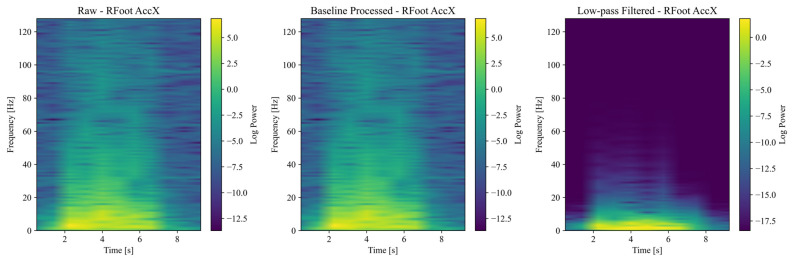
Spectrograms generated from accelerometer signals.

Accelerometer and gyroscope signals collected from IMUs were transformed into time–frequency spectrograms to serve as input for deep learning models. The Short-Time Fourier Transform (STFT) was applied to the raw, preprocessed and filtered IMU signals using a sampling frequency of 256 Hz and a Hanning window of size 256 samples with 50% overlap, generating a time–frequency representation of the signal’s power spectral density. To improve the visibility of spectral features, the power spectral density was log-transformed. Each spectrogram was saved as a color image (RGB) with axes and labels removed to ensure a clean input for the convolutional neural network (CNN). Figure 5 shows example spectrograms generated from accelerometer and gyroscope signals from the foot segment. Despite the short walking distances (~4 m), we included the entire recorded sequence, including acceleration and deceleration phases, to mirror a realistic clinical setup and maintain trial consistency. Clinical studies indicate that relevant movement features can emerge during transitional gait phases.

#### 2.1.2. Data Partitioning and Leakage Control

All experiments used a subject-wise split: the three walking trials recorded for a given participant were always assigned to the same training, validation or test partition to avoid data leakage. The resulting fold sizes were 106/13/14 subjects (training/validation/test).

### 2.2. Clinical Data

Clinical data contains the evaluation of participants based on frailty assessment methodology and clinical features that were imported from an Excel spreadsheet and then processed by normalization. Clinical features are organized into grip strength, physical activity, timed tests, balance tests, gait assessment, demographics and gait parameters (Table 1). Descriptive statistics are reported as mean ± standard deviation, and differences among the three frailty categories for each clinical or gait-related feature were evaluated with an omnibus test. When the omnibus test reached significance (threshold *p* < 0.05), post hoc pair-wise comparisons were carried out (Bonferroni-adjusted independent-samples *t*-tests after ANOVA). Principal Component Analysis (PCA) was conducted within each feature group to reduce dimensionality while maintaining 95% of the variance.

### 2.3. Subject-Based Data Splitting

To ensure realistic generalization and prevent data leakage between training and testing, a subject-wise data split was applied. In total, 80% of participants were divided into training, 10% to validation and 10% to testing subsets. There were 108 spectrograms per subject (3 axes of accelerometer and 3 axes of gyroscope per sensor, 6 sensors on 6 lower leg segments and 3 trials), totaling 14364 samples per dataset. Each spectrogram file name encoded the participant ID, sensor location (thigh, shank, foot), side (left, right), and signal channel. These IDs were used to group all spectrograms belonging to the same participant. Stratified random sampling based on frailty categories (Frail, PreFrail and NoFrail) was implemented to ensure proportional allocation by frailty class, thereby preserving the original class distribution within each subset. Participants were independently assigned to training, validation and test subsets, ensuring that all spectrograms from a specific participant were grouped within the same subset.

### 2.4. Deep Learning Model Architecture

The image branch (Figure 1) consisted of a pre-trained ResNet-50 convolutional neural network (CNN), which was pre-trained on ImageNet, comprising 50 convolutional layers organized into five main stages. ResNet-50 was chosen for its demonstrated effectiveness in capturing intricate spatial hierarchies, including those in non-natural image domains such as spectrograms [54,55]. Its deep residual architecture facilitates the efficient learning of time–frequency patterns that indicate gait abnormalities and frailty-specific spectral signatures. The initial convolutional layers employed a 7 × 7 kernel size, followed by multiple 3 × 3 kernels in subsequent residual blocks. Early convolutional layers were frozen, while layers 3 and 4 were fine-tuned during training to adapt to spectrogram data. The final fully connected layer of ResNet-50 was replaced with a customized classification head that features batch normalization, dropout regularization (with rates of 0.2 and 0.3), and ReLU activation functions to reduce overfitting on relatively small datasets and enhance model generalizability. The clinical branch consisted of a two-layer feedforward neural network that processed the reduced clinical feature vector obtained after principal component analysis (PCA). This network consisted of an input layer (with the same number of neurons as the PCA-reduced feature set size), followed by hidden layers with 64 and 32 neurons, each activated by ReLU and regularized with dropout (with a rate of 0.3). Outputs from both branches were concatenated and passed through a fully connected fusion layer containing 256 neurons, a ReLU activation function and dropout regularization (rate of 0.5). The spectrogram and clinical features were concatenated and passed through a fully connected fusion layer, followed by a SoftMax output layer for final classification into the three frailty categories.

### 2.5. Training Process

Model training and evaluation were conducted using GPU acceleration with CUDA. Due to imbalanced class sizes, class weights were assigned inversely proportional to the frequency of each class, i.e., 1.2 for Frail, 0.7 for PreFrail and 1.3 for NoFrail. During the training, the cross-entropy loss function was used as the objective, and the Adam optimizer was utilized with a learning rate of 0.0005. Early stopping with a patience of 5 epochs was applied to avoid overfitting, monitoring the validation accuracy. A batch size of 32 was employed. Each of the three spectrogram datasets—raw, baseline-processed and low-pass filtered—was trained independently to assess the effect of signal conditioning on model performance. This resulted in three separate trained models, each optimized under identical hyperparameters and evaluation protocols. A 5-fold Stratified Cross-Validation was used to ensure balanced train, validation and test splits, maintaining proportional class representation.

### 2.6. Evaluation Metrics

Assessment of classification accuracy is performed at both the image and subject scales. Model performance was assessed at two levels:

- Spectrogram-Level Evaluation. Classification accuracy, confusion matrix, sensitivity, specificity and balanced accuracy were computed at the individual spectrogram level.
- Subject-Level Evaluation. Each participant was assigned a final predicted label using a majority vote across all their spectrograms. Subject-level confusion matrices and accuracy were also computed to reflect better real-world performance, where clinical decisions would be based on whole-subject classification.

To determine whether the observed differences in classification accuracy and other metrics among the raw, baseline and low-pass filtered methods were statistically significant, a Friedman test was performed on the results obtained from 5-fold cross-validation. Each fold’s performance metric (accuracy, F1-score, etc.) was treated as a repeated measure. Where the Friedman test indicated significance (*p* < 0.05), a post hoc test (e.g., Nemenyi test) was applied to identify which methods significantly differed.

Separate training on clinical data was performed to gauge the benefit of the clinical features only, and accuracy and macro-F1 were averaged over the three cross-validation folds (same splits as in the main experiment).

Additionally, to assess the possible effects of data leakage, an analysis was conducted by comparing three evaluation scenarios: (a) subject-wise classification (where all spectrograms from a subject are included in either the training, validation or test sets); (b) random split classification (where spectrograms from the same subject could appear in several dataset partitions) and (c) cross-evaluation classification (validating across different subjects).

## 3. Results

This study evaluated the classification performance of raw, processed, and low-pass filtered IMU signals combined with clinical features, as well as an alternative approach based solely on spectrogram representations of IMU-derived gait data. The primary objective of this study was to evaluate whether raw or minimally processed IMU signals, represented as spectrograms, could effectively differentiate between Frail, PreFrail, and NoFrail individuals. The classification performance was assessed using precision, recall, F1-score and overall accuracy, comparing the ability of different data processing techniques to distinguish between Frail, PreFrail and NoFrail individuals.

Table 2 presents the classification performance across these datasets using a multimodal classification model.

The findings reveal that a combination of raw IMU data and clinical features reached an average accuracy of 85.19%, highlighting that even minimally processed signals hold valuable information for classifying frailty. The precision and recall metrics for the Frail and PreFrail categories were notably high (0.945 and 0.881 for precision, 0.925 and 0.811 for recall, respectively), implying that unprocessed sensor data alone forms a solid foundation for classification. Nonetheless, the NoFrail category demonstrated lower precision (0.703) and recall (0.825), pointing to a higher chance of incorrect classification for NoFrail individuals. The macro-averaged F1-score for this method was 0.842, indicating a balanced performance across all categories. Implementing preprocessing strategies on IMU data, specifically stripping the gravitational element and normalizing signals, led to enhanced classification efficacy. The baseline processed IMU dataset saw a rise in accuracy to 88.15%, with significant gains in identifying Frail individuals, where the F1-score went up to 0.995. The PreFrail category experienced a moderate rise in F1-score (0.879), while NoFrail classification remained unchanged (F1-score = 0.754). These results indicate that preprocessing signals boost deep learning models’ capability to differentiate between frailty categories, especially for the most critical cases. Further enhancement via low-pass filtering of IMU signals kept the overall classification accuracy at 88.15%, with slight variations in the performance of individual categories. The F1-score for NoFrail classification slightly improved to 0.773, showing that filtering reduced noise and improved the model’s accuracy in identifying NoFrail individuals. However, a slight drop in Frail classification (F1-score = 0.979) suggests that removing high-frequency elements could have removed essential gait-related data for the most severe frailty cases. Regardless, the macro-averaged F1-score stayed consistent at 0.877, affirming the overall stability of both processing techniques.

The statistical significance tests using the Friedman test were performed on macro-level performance (averaged across all classes) and per-class metrics for Frail, PreFrail, and NoFrail. Recall in the Frail class had the largest Friedman statistic (3.50) but did not reach significance (*p* = 0.1738). Precision and F1 also showed no significant differences (*p* = 0.9460 and *p* = 0.8187, respectively). No metric demonstrated significance in the PreFrail class (all *p*-values > 0.05). Similarly, Friedman tests showed non-significant differences for precision, recall and F1-score (highest statistic = 1.60) in the NoFrail class.

To further evaluate the feasibility of spectrogram-based frailty classification, models were trained solely on time–frequency representations of IMU signals without explicit clinical features. The dataset was structured as follows: a subject-level dataset containing *n* = 106 for training, *n* = 13 for validation, and *n* = 14 for testing subjects; a spectrogram-level dataset containing Training *n* = 11448, Validation *n* = 1404, and Test *n* = 1512 spectrogram images.

**Figure 6:**
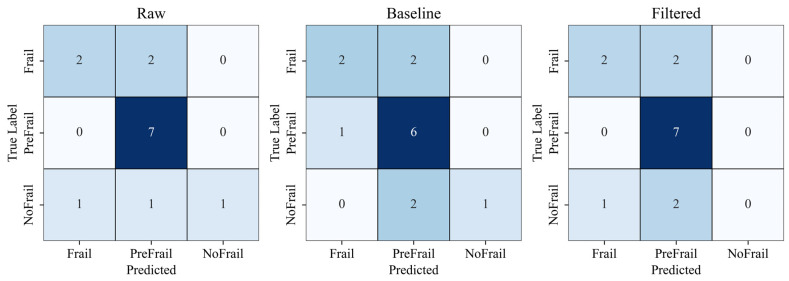
Subject-level classification confusion matrices comparing raw, processed, and filtered spectrogram models. Color intensity indicates classification accuracy (darker colors represent higher values).

Using a strict subject-wise split, the Raw spectrogram model reached 71.43% accuracy, substantially higher than the Processed (64.29%) and Filtered spectrogram models (64.29%) (Figure 6). These results suggest that aggressive filtering may remove subtle high-frequency gait components informative for frailty staging.

**Figure 7:**
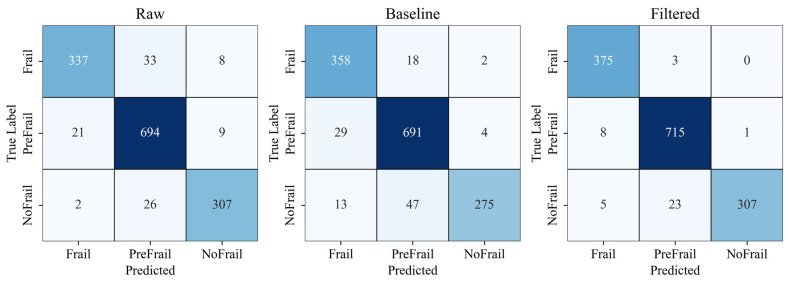
Spectrogram-level classification confusion matrices comparing raw, processed and filtered spectrogram models. Color intensity indicates classification accuracy (darker colors represent higher values).

After analyzing the top-performing spectrogram models through confusion matrix analysis, the following test accuracies were observed: Raw spectrograms achieved 93.11%, Processed spectrograms reached 97.22%, and Low-pass filtered spectrograms attained 92.14% (Figure 7).

The numerical performance of the subject-level and spectrogram-level splits of spectrogram-only models is summarized in Table 3.

These findings demonstrate that deep learning models trained solely on spectrogram data can achieve high accuracy rates. Notably, the processed spectrograms provided the highest classification performance with 97.22% accuracy. This indicates that the time–frequency representations of IMU signals are effective for classifying frailty, even without the inclusion of specifically designed clinical features.

The clinical feature block achieved an accuracy of 65.38%, lower than the 71.4% accuracy obtained using raw spectrograms. Combining these two inputs increased the accuracy to 72.75% (Δ = 1.35%).

**Figure 8:**
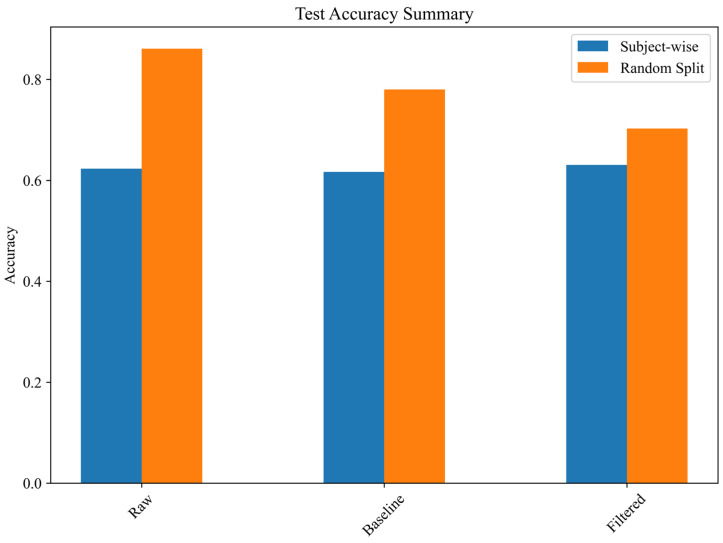
Effect of data leakage on test accuracy.

Data leakage analysis shows that random splitting artificially inflates performance by 15.8% (Figure 8).

Results confirm a significant overestimation of classification performance when random splits are used instead of subject-wise separation. In particular, the random split test accuracy for the raw spectrogram model was 86.08%, whereas its subject-wise accuracy was only 62.31%, revealing an overestimation factor of 1.38. This suggests that models trained on spectrograms may memorize subject-specific patterns rather than generalizable gait characteristics, leading to inflated performance in cross-validation scenarios that do not enforce subject separation. The findings indicate that random distribution of spectrograms across the training, validation, and test sets results in exaggerated classification performance, likely due to overfitting and feature redundancy when images from the same subject are included in multiple partitions.

## 4. Discussion

The findings of this research offer insights into the possibility of categorizing frailty status using deep learning applied to IMU-derived gait signals and spectrogram-based representations. The results indicate that both raw and processed IMU signals have an adequate ability to differentiate among Frail, PreFrail, and NoFrail individuals. However, signal preprocessing is essential for improving classification performance, especially for detecting Frail individuals. Comparing raw, baseline processed and low-pass filtered IMU signals revealed that preprocessing increased classification accuracy from 85.19% (raw) to 88.15% (processed and filtered). Spectrogram-based classification accuracy is similar to the time-series classification using the InceptionTime model (81%); however, in a study by Amjad et al., they did not perform classification on a subject-based level [46]. Arshad achieved similar classification performance by combining STFT and GAF representations of IMU gait signals of frailty subjects [43,44]. Although the processed and baseline filtered methods produced slightly higher average performance metrics than raw in some cases (e.g., accuracy, F1-score), the observed differences did not reach statistical significance.

The most significant enhancement was noted in the Frail category, where the F1-score rose from 0.933 (raw) to 0.995 (processed). This implies that preprocessing boosts the model’s capability to detect movement deficits linked to frailty, likely by decreasing sensor noise and eliminating irrelevant signal variations. Nonetheless, classification performance for NoFrail individuals was consistently lower across all datasets (F1-score: 0.752 for raw, 0.754 for processed, 0.773 for filtered), suggesting that distinguishing healthy older adults from PreFrail individuals is still difficult. This is consistent with previous studies that showed frailty transitions are gradual rather than binary, and gait changes may be more subtle in early frailty stages [1,2]. Looking at the clinical features alone, in our previous study we have determined that gait speed was the most sensitive parameter for the identification of frailty and stride time was sensitive for discriminating against PreFrail or Frail from NoFrail [32]. Moreover, increased double support time was expressed in PreFrail and Frail subjects. Further optimization, such as combining biomechanical parameters or incorporating additional motion-based descriptors, could enhance NoFrail classification. Notably, low-pass filtering did not significantly outperform baseline processing, indicating that eliminating high-frequency components does not necessarily improve classification performance. While filtering slightly enhanced NoFrail classification, it somewhat reduced Frail classification accuracy, possibly due to the loss of some high-frequency gait features. This underscores the significance of preserving signal integrity in the design of gait classification models. To determine whether performance differences among the raw, baseline processed, and low-pass filtered methods were statistically significant, Friedman tests on the classification metrics (accuracy, precision, recall and F1-score) were conducted. None of the methods showed statistically superior macro-level and per-class metrics of accuracy, precision, recall or F1-score. These findings suggest that the choice of signal preprocessing does not systematically alter classification performance when combined with clinical features, at least under our dataset and experimental conditions.

The assessment of spectrogram-based deep learning models, especially combining them with clinical features, looks promising as an alternative strategy for classifying frailty. The models relying solely on spectrograms demonstrated high accuracy in classification, with the top-performing model (processed spectrograms) attaining an accuracy of 97.22%; however, due to the still small subject number for machine learning applications, there is a risk of overfitting and poor generalization. Time–frequency representations adequately capture pertinent gait features necessary for frailty classification, establishing them as a feasible alternative to time-series methods. Nonetheless, a key issue in spectrogram-based classification is data leakage, where spectrograms from the same individual might be split across training, validation, and test sets. Our analysis showed that random spectrogram splitting artificially increased test accuracy, causing overclassification bias. For instance, the test accuracy for raw spectrogram classification rose from 62.31% (subject-wise split) to 86.08% (random split), resulting in an overestimation factor of 1.38. This underscores the need for subject-wise data separation to ensure models can generalize to new individuals instead of memorizing individual-specific patterns. Despite this challenge, subject-wise spectrogram classification still produced encouraging results, with raw spectrograms achieving 71.43% accuracy. Combining with clinical features only slightly increases accuracy to 72.75 %, indicating a small but non-decisive gain. This implies that IMU-derived spectrograms alone offer valuable insights for frailty assessment, even without clinical features. Future research may investigate other spectrogram transformations, such as wavelet decomposition, recurrence plots or deep feature embeddings, to enhance classification robustness.

The results of this research have significant implications for the screening of frailty and its practical application in clinical settings. The capability to identify frailty with minimally processed IMU data endorses the potential for automated, wearable-based assessments in both clinical and home environments. In contrast to conventional frailty evaluations that depend on clinical assessments or gait laboratories, our method shows that machine learning can derive meaningful features from raw sensor data, which may lessen the need for manual feature engineering. Furthermore, the high accuracy of spectrogram-based models indicates that deep learning can effectively interpret time–frequency patterns in IMU signals. This could lead to the incorporation of spectrogram-based frailty assessment into mobile health applications or remote patient monitoring systems. However, our results also highlight the importance of stringent evaluation protocols to maintain subject-wise separation, thereby preventing the overestimation of classification performance.

There are multiple limitations to consider. First, the dataset utilized in this research was gathered in controlled laboratory settings, which might not accurately reflect real-world gait evaluations where external factors, such as uneven terrain and obstacles, add further variability. Second, treating the three trials per participant as separate inputs enlarges the training set and exposes the network to stride-to-stride variability. At the same time, the subject-wise split guarantees that no information from an individual leaks into the validation or fold. The subject-wise partition and inverse-frequency class weighting in the loss function prevent information leakage but cannot substitute for a larger, more diverse cohort. Future studies should therefore enroll more participants to improve external validity. Third, we analyzed only the accelerometer and gyroscope channels. Future investigations could integrate additional sensor types, like force sensors or electromyography (EMG), to capture more detailed movement characteristics. Fourth, examining self-supervised learning or transformer-based models could improve classification accuracy by capturing extended gait dynamics. Finally, despite the high performance of frailty classification, the ability to clinically interpret deep learning models remains an issue. Upcoming work might prioritize explainable AI approaches, like saliency maps or attention mechanisms, to determine which spectrogram areas or IMU signal patterns are most influential in classification decisions. In future research, validating models on larger [46,56] and maybe more diverse cohorts would be beneficial to ensure reliability and applicability. In parallel, exploiting advanced time–frequency methods could also help to capture subtle gait adjustments and patterns, thus enhancing early-stage frailty detection. Finally, sensitivity analyses that rank the contribution of each sensor location could identify a minimal, patient-friendly IMU configuration and reveal segment-specific gait signatures of frailty.

## 5. Conclusions

This research demonstrates that preprocessing signals enhance the precision of frailty assessments, particularly in identifying Frail individuals. Although low-pass filtering slightly benefits NoFrail classification, it may filter out significant high-frequency gait details. Raw IMU-derived spectrograms enabled a subject-wise frailty classification accuracy of 71.4%, confirming that minimally processed wearable signals contain discriminative gait patterns. Additional pre-processing did not improve and occasionally reduced performance. While augmenting the model with clinical features can raise the headline accuracy, that benefit vanishes when strict subject separation is applied, underscoring the importance of leakage-free evaluation protocols. Taken together, our results show that a fully passive, wearable-only pipeline is feasible for frailty screening, and that careful control of signal preprocessing is critical for reliable deployment. These findings highlight the promise of IMU-based frailty evaluations in clinical settings.

## Figures and Tables

**Table 1:** Clinical evaluation features.

Feature	Frail	PreFrail	NoFrail	*p*-Value
Grip strength				
Right 1 Grip Force	20.51 ± 8.82	27.58 ± 8.77	28.35 ± 7.58	0.00013
Left 1 Grip Force	20.31 ± 9.45	26.55 ± 9.83	27.90 ± 7.64	0.00142
Right 2 Grip Force	20.54 ± 9.09	28.19 ± 9.08	30.13 ± 7.39	0.00002
Left 2 Grip Force	20.57 ± 9.33	27.46 ± 10.05	28.71 ± 7.41	0.00049
Right 3 Grip Force	20.71 ± 9.05	28.73 ± 8.79	30.13 ± 7.58	0.00001
Left 3 Grip Force	20.60 ± 9.45	27.61 ± 9.59	28.58 ± 7.73	0.00039
Max Grip Force	23.06 ± 8.77	31.22 ± 8.73	31.81 ± 7.91	0.00001
Mobility				
TUG time	15.67 ± 4.89	12.69 ± 4.53	7.64 ± 1.84	0.00000
Tinneti Test score	20.54 ± 3.90	22.28 ± 3.49	25.48 ± 2.71	0.00000
TT balance	11.40 ± 2.39	12.24 ± 1.99	14.23 ± 1.91	0.00000
TT gait	9.06 ± 2.04	10.03 ± 1.87	11.16 ± 1.37	0.00004
BERG balance	37.77 ± 6.81	40.91 ± 5.88	49.16 ± 7.37	0.00000
Dynamic Gait Index	14.26 ± 4.22	16.15 ± 3.50	19.32 ± 3.34	0.00000
Anthropometrics				
Age	78.80 ± 7.50	74.00 ± 8.47	73.19 ± 6.34	0.00479
Height (cm)	160.86 ± 6.43	165.79 ± 9.59	162.74 ± 9.60	0.02418
Weight (kg)	68.43 ± 15.96	75.85 ± 15.69	76.13 ± 16.27	0.00604
BMI	26.47 ± 6.29	27.68 ± 5.67	28.72 ± 5.52	0.29165
Spatiotemporal Gait Parameters				
Gait Velocity (m/s)	0.51 ± 0.13	0.63 ± 0.13	0.97 ± 0.16	0.00000
Gait Time (s)	8.42 ± 2.51	6.69 ± 1.61	4.25 ± 0.79	0.00000
Right Swing (s)	0.50 ± 0.08	0.51 ± 0.08	0.44 ± 0.06	0.00025
Left Swing (s)	0.53 ± 0.07	0.54 ± 0.06	0.48 ± 0.07	0.00007
Right Stance (s)	0.97 ± 0.21	0.84 ± 0.13	0.63 ± 0.11	0.00000
Left Stance (s)	0.93 ± 0.21	0.81 ± 0.13	0.59 ± 0.10	0.00000
Right Stride (s)	1.47 ± 0.25	1.35 ± 0.16	1.07 ± 0.15	0.00000
Left Stride (s)	1.46 ± 0.23	1.35 ± 0.16	1.06 ± 0.15	0.00000
Double Support (s)	0.21 ± 0.08	0.15 ± 0.07	0.08 ± 0.04	0.00000
Cadence	84.54 ± 12.98	91.32 ± 11.83	116.75 ± 13.17	0.00000
Step Time (s)	0.73 ± 0.13	0.67 ± 0.08	0.52 ± 0.06	0.00000
Right Swing (%)	34.51 ± 5.02	37.64 ± 4.38	41.28 ± 3.30	0.00000
Left Swing (%)	36.55 ± 4.71	40.35 ± 4.07	44.98 ± 3.50	0.00000
Right Stance (%)	65.49 ± 5.02	62.36 ± 4.38	58.72 ± 3.30	0.00000
Left Stance (%)	63.45 ± 4.71	59.65 ± 4.07	55.02 ± 3.50	0.00000

**Table 2:** Classification performance results.

Class	Precision	Recall	F1-Score	Support	Average Accuracy (%)
Raw IMU data + clinical features					
Frail	0.945 ± 0.075	0.925 ± 0.081	0.933 ± 0.070	756	
PreFrail	0.881 ± 0.077	0.811 ± 0.108	0.841 ± 0.081	1512	
NoFrail	0.703 ± 0.101	0.825 ± 0.099	0.752 ± 0.072	648	85.19
macro avg	0.843 ± 0.071	0.854 ± 0.071	0.842 ± 0.071	2916	
weighted avg	0.858 ± 0.069	0.844 ± 0.073	0.845 ± 0.073	2916	
Baseline processed IMU data + clinical features					
Frail	0.993 ± 0.007	0.996 ± 0.006	0.995 ± 0.003	756	
PreFrail	0.907 ± 0.037	0.854 ± 0.058	0.879 ± 0.047	1512	
NoFrail	0.715 ± 0.106	0.801 ± 0.070	0.754 ± 0.087	648	88.15
macro avg	0.871 ± 0.045	0.884 ± 0.043	0.876 ± 0.045	2916	
weighted avg	0.886 ± 0.041	0.879 ± 0.045	0.881 ± 0.044	2916	
Low-pass filtered IMU data + clinical features					
Frail	0.980 ± 0.035	0.979 ± 0.041	0.979 ± 0.024	756	
PreFrail	0.915 ± 0.042	0.846 ± 0.056	0.878 ± 0.043	1512	
NoFrail	0.719 ± 0.078	0.838 ± 0.066	0.773 ± 0.070	648	88.15
macro avg	0.871 ± 0.041	0.888 ± 0.041	0.877 ± 0.041	2916	
weighted avg	0.888 ± 0.038	0.879 ± 0.042	0.881 ± 0.041	2916	

**Table 3:** Classification performance results of spectrogram-only models.

Spectrograms	Accuracy (%)	Macro Precision	Macro Recall	Macro F1
Subject-level				
Raw	71.43	0.74	0.68	0.70
Baseline	64.29	0.67	0.62	0.63
Filtered	64.29	0.68	0.61	0.63
Spectrogram-level				
Raw	86.08	0.86	0.85	0.85
Baseline	97.22	0.97	0.97	0.97
Filtered	92.14	0.92	0.92	0.92

## Data Availability

Dataset available on request from the authors.

## Abbreviations

The following abbreviations are used in this manuscript:

IMU	Inertial Measurement Unit
CNN	Convolutional Neural Network
STFT	Short-Time Fourier Transform
